# Impact of Social Determinants of Health on Hepatocellular Carcinoma Surveillance Receipt and Early Detection

**DOI:** 10.1016/j.cgh.2025.09.037

**Published:** 2025-10-10

**Authors:** MOHAMMED AL-HASAN, NICOLE E. RICH, SRUTHI YEKKALURI, PATRICIA D. JONES, ADAM YOPP, AMIT G. SINGAL

**Affiliations:** Department of Internal Medicine, UT Southwestern Medical Center, Dallas, Texas; Department of Internal Medicine, UT Southwestern Medical Center, Dallas, Texas, *and* Parkland Health, Dallas, Texas; Department of Internal Medicine, UT Southwestern Medical Center, Dallas, Texas; Division of Digestive Health and Liver Diseases, Department of Internal Medicine, University of Miami, Miami, Florida, *and* Sylvester Comprehensive Cancer, Center Miami, Florida; Department of Surgery, UT Southwestern Medical Center, Dallas, Texas; Department of Internal Medicine, UT Southwestern Medical Center, Dallas, Texas, *and* Parkland Health, Dallas, Texas

Hepatocellular carcinoma (HCC) is a leading cause of cancer death in patients with cirrhosis, with a disproportionate burden among racial and ethnic minority populations.^1^ HCC surveillance is associated with improved early-stage detection and overall survival but remains underutilized.^2,3^ Social determinants of health (SDOH) and patient health beliefs are associated with failures in the cancer care continuum in other cancers, but there are fewer studies in patients with HCC.^4^ We investigated the association between SDOH and surveillance and early-stage detection in patients with HCC from the United States.

The ME-HCC (Multi-Ethnic Hepatocellular Carcinoma) cohort enrolled adult patients diagnosed with HCC between September 2018 and July 2023 from 4 health systems: UT Southwestern, Parkland Health, University of Miami, and Jackson Memorial Hospital.^5^ Patients with previously treated HCC, uncontrolled hepatic encephalopathy, and language other than English or Spanish were excluded. The study was approved by the institutional review boards at UT Southwestern and University of Miami.

Demographic and clinical features were extracted from electronic medical records. Details of survey administration and results have been reported.^6^ Patients completed a baseline survey prior to HCC treatment, in English or Spanish, and a follow-up survey ~2 to 6 months later. The baseline survey collected patients’ demographics, household income, employment status, and educational attainment. It incorporated validated instruments to assess medical mistrust (Group-Based Medical Mistrust Scale), health literacy (Chew Health Literacy Assessment), and the shortened Everyday Discrimination Scale. The follow-up survey assessed health locus of control, patient-provider communication, and barriers to care. The Chew Health Literacy Assessment was dichotomized, with scores ≤9 indicating limited health literacy. Other scales were analyzed as continuous variables using a 5-point Likert scale ranging from “strongly disagree” to “strongly agree.” If ≤25% of responses for an SDOH scale were missing, we used person-mean imputation based on other responses from the individual’s scale. Conversely, scales with >25% of data missing were excluded from analyses. See the Supplementary Methods for details.

We performed univariable and multivariable logistic regression analyses to identify factors associated with surveillance receipt and early-stage detection. Surveillance was defined as an ultrasound, multiphase computed tomography, and/or contrast-enhanced magnetic resonance imaging in 3 to 9 months prior to HCC diagnosis. Early-stage HCC was defined per the Milan criteria. Multivariable models included variables deemed clinically important a priori including race/ethnicity, liver etiology, Child-Pugh class, site, income, insurance, and SDOH scales. We performed separate analyses among patients who completed follow-up surveys. Statistical significance was defined as *P* < .05. Analyses were performed using SAS version 9.4 (SAS Institute).

Of 1245 eligible patients, 833 (66.9%) completed a baseline survey, and of those, 495 (59.4%) completed follow-up surveys (Supplementary Table 1). The median age of respondents was 64.2 years, and 631 (75.8%) were men. The cohort included 382 (45.9%) Hispanic, 299 (35.9%) non-Hispanic White, and 118 (14.2%) Black individuals. More than half (55.4%) had Child-Pugh A cirrhosis.

Only 337 (40.5%) patients received HCC surveillance. In multivariable analysis (Table 1), surveillance was associated with alcohol-associated etiology (vs hepatitis C: odds ratio [OR], 1.63; 95% confidence interval [CI], 1.05–2.55), lack of insurance (vs Medicare: OR, 0.36; 95% CI, 0.13–0.91), and adequate health literacy (OR, 1.42; 95% CI, 1.01–2.00). Other survey measures failed to have significant associations, including medical mistrust (OR, 1.26; 95% CI, 0.90–1.76), patient-provider communication (OR, 1.02; 95% CI, 0.97–1.07), and barriers to care (OR, 1.01; 95% CI, 0.95–1.08).

Less than half of patients (47.8%) were detected within the Milan criteria. In multivariable analysis, early-stage detection was positively associated with surveillance receipt (OR, 3.14; 95% CI, 2.25–4.42), while it was negatively associated with Black race (OR, 0.50; 95% CI, 0.27–0.90), Child-Pugh B (OR, 0.63; 95% CI, 0.43–0.92), and age >65 years (OR, 0.64; 95% CI, 0.42–0.98) (Table 1). Most patients (64.1%) with surveillance had early-stage HCC, compared with only 37.1% of those without surveillance. Similarly, early-stage HCC was observed in 49.8% of patients with Child-Pugh A vs 45.1% with Child-Pugh B cirrhosis, and 44.6% of patients >65 years of age vs 50.4% of those ≤65 years of age. In multivariable analysis among patients who completed follow-up surveys, better patient-provider communication (continuous) was associated with early-stage HCC (OR, 1.08; 95% CI, 1.03–1.14).

Overall, our findings align with literature that surveillance receipt is the strongest predictor of early-stage detection but continues to be underused in practice.^2,3^ Our study adds to existing literature by assessing associations with several SDOH measures and patient health beliefs. We found that health literacy and patient-provider communication were associated with surveillance and early-stage presentation, highlighting the importance of patient engagement and education for timely diagnosis.^7^ Effective communication fosters trust, promotes shared decision making, and encourages engagement with the healthcare system. However, we failed to find a significant association between other SDOH and surveillance receipt, suggesting that other factors, such as system-level barriers or provider knowledge, also play a large role in surveillance completion.^8,9^ Addressing barriers through patient navigation, provider education, and system-level interventions may help improve surveillance uptake.^3^

We also observed racial and ethnic differences in outcomes. Whereas Black patients were less likely to present at an early stage, Hispanic ethnicity was associated with trend toward increased early-stage HCC detection. This discordant finding compared with existing literature may be influenced by differences in healthcare structure at health systems in our study, with each having large Hispanic populations and implementing measures such as access to Spanish-speaking staff, which can mitigate barriers to care.^10^ Future studies using causal mediation analyses, including SDOH and health beliefs, may provide insights into sociodemographic disparities.

Study limitations include missing data, ascertainment bias, and measurement bias given its retrospective nature. Second, self-reported survey data are prone to recall bias, nonresponse bias, and social desirability bias, particularly for measures such as medical mistrust and health literacy. Third, our results may not generalize to other populations or settings, particularly outside the United States.

In conclusion, surveillance is associated with early HCC detection but remains underused in practice. Beyond previously reported correlates, we observed unique associations including health literacy being associated with surveillance receipt and patient-provider communication being associated with early-stage HCC presentation. Studies should identify intervention targets to increase HCC surveillance receipt to improve early HCC detection.

## Supplementary Material

1

Note: To access the supplementary material accompanying this article, visit the online version of *Clinical Gastroenterology and Hepatology* at www.cghjournal.org, and at https://doi.org/10.1016/j.cgh.2025.09.037.

## Figures and Tables

**Table 1.** Factors Associated With HCC Surveillance and Early HCC Detection in Multivariable Analyses

Variable	HCC surveillance^a^	Early HCC detection^a^

Age (Ref: ≤65 y)		
> 65 y	0.75 (0.50–1.13)	0.64 (0.42–0.98)
Sex (Ref: female)		
Male	0.78 (0.52–1.15)	0.69 (0.46–1.04)
Race/ethnicity (Ref: White)		
Hispanic	1.35 (0.91–2.01)	1.34 (0.89–2.03)
Black	0.92 (0.52–1.62)	0.50 (0.27–0.90)
Other	1.37 (0.61–3.04)	0.86 (0.38–1.94)
Liver etiology (Ref: HCV)		
Alcohol related	1.63 (1.05–2.55)	0.95 (0.60–1.52)
Hepatitis B infection	0.93 (0.36–2.29)	0.46 (0.17–1.19)
MASLD	1.48 (0.91–2.40)	0.63 (0.38–1.03)
Other	1.65 (0.87–3.18)	0.61 (0.30–1.22)
Child-Pugh class (Ref: Child-Pugh A)		
B	1.15 (0.81–1.65)	0.63 (0.43–0.92)
C	1.10 (0.65–1.85)	0.67 (0.38–1.15)
Annual household income (Ref: <$20,000)		
$20,000-$50,000	0.89 (0.52–1.50)	1.62 (0.94–2.83)
>$50,000	0.75 (0.42–1.34)	1.50 (0.82–2.74)
Insurance (Ref: Medicare)		
Private/ACA exchange	0.89 (0.57–1.40)	0.86 (0.54–1.37)
Medicaid	1.05 (0.46–2.38)	0.71 (0.29–1.72)
Parkland Financial Assistance/Jackson card	0.69 (0.36–1.32)	0.55 (0.27–1.10)
Other	0.72 (0.38–1.34)	0.86 (0.45–1.65)
Uninsured	0.36 (0.13–0.91)	0.95 (0.37–2.44)
GBMMS (continuous)	1.26 (0.90–1.76)	1.17 (0.82–1.66)
Chew Health Literacy (Ref: ≤9)		
>9	1.42 (1.01–2.00)	1.07 (0.75–1.53)
EDS (continuous)	1.02 (0.95–1.09)	0.98 (0.91–1.05)
Surveillance receipt		3.14 (2.25–4.42)
Patient-provider communication (continuous)^b^	1.02 (0.97–1.07)	1.08 (1.03–1.14)
Barriers to care^b^	1.01 (0.95–1.08)	1.01 (0.94–1.08)

Values are odds ratio (95% confidence interval).

ACA, Affordable Care Act; EDS, everyday discrimination score; GBMMS, Group-Based Medical Mistrust Score; HCC, hepatocellular carcinoma; HCV, hepatitis C virus; MASLD, metabolic dysfunction-associated steatotic liver disease.

a Models are additionally adjusted for study site.

b Patient-provider communication and barriers to care were assessed in follow-up surveys and thus were available in a subset of patients.
