# Supplementary material for: Impact of Social Determinants of Health on Hepatocellular Carcinoma Surveillance Receipt and Early Detection
Source: Clin Gastroenterol Hepatol. Author manuscript; Available in PMC 2026 Aug 12. (PMC13461308; doi:10.1016/j.cgh.2025.09.037)
Supplement: Supplementary file 1 [file NIHMS2198526-supplement-1.pdf]

Supplementary Methods

HCC diagnoses were consistent with Liver Imaging Reporting and Data System (LI-RADS) criteria.

Race and ethnicity were self-reported and categorized as non-Hispanic White (White), non-Hispanic Black (Black), Hispanic (from any race), or Other. Liver disease etiology was categorized hierarchically into hepatitis C virus, hepatitis B virus (HBV), alcohol-associated liver disease (ALD), other (e.g., hemochromatosis), metabolic dysfunction-associated steatotic liver disease (MASLD), or unknown liver disease. Liver disease severity was assessed using the Child Pugh class, and patient performance status was captured using the Eastern Cooperative Oncology Group (ECOG) scale. Insurance status was categorized as Medicare, Medicaid, private insurance/affordable care act (ACA) exchange, Parkland financial assistance/Jackson card (i.e., medical subsidy plans which cover HCC surveillance at Parkland and Jackson), other, or no insurance. Tumor characteristics were also extracted including the number of HCC lesions, maximum tumor diameter, vascular invasion, and distant metastases at diagnosis.

Cronbach's alpha was 0.87 for Group Based Medical Mistrust Scale[1], 0.73 for Chew Health Literacy Assessment[2], and 0.77 for the shortened Everyday Discrimination Scale.[3, 4] Overall, less than 5% of survey data from respondents were missing. Person-mean imputation replaces missing items for an individual by the mean of the individual's observed items in the scale and is less subject to creating bias than mean imputation (based on entire cohort).[5]

Supplementary References

1. Shelton RC, Winkel G, Davis SN, et al. Validation of the group-based medical mistrust scale among urban black men. J Gen Intern Med 2010;25:549-55.
2. Dageforde LA, Cavanaugh KL, Moore DE, et al. Validation of the Written Administration of the Short Literacy Survey. J Health Commun 2015;20:835-42.
3. Al-Hasan M, Rich NE, Figueroa G, et al. Racial and ethnic differences in social determinants of health among patients with HCC. Hepatol Commun 2025;9.
4. Sternthal MJ, Slopen N, Williams DR. RACIAL DISPARITIES IN HEALTH: How Much Does Stress Really Matter? Du Bois Rev 2011;8:95-113.
5. Rezvan PH, Comulada WS, Fernandez MI, et al. Assessing Alternative Imputation Strategies for Infrequently Missing Items on Multi-item Scales. Commun Stat Case Stud Data Anal Appl 2022;8:682-713.

Supplementary Table 1: Demographic and clinical characteristics of patients who completed baseline surveys (n=833)

Characteristic	Frequency (%)
**Site**	
Jackson	101 (12.1)
Parkland	270 (32.4)
UT Southwestern	285 (34.2)
U Miami	177 (21.3)
**Age, years***	64.2 (59.0-69.9)
**Gender**	
Men	631 (75.8)
**Race/Ethnicity**	
Non-Hispanic White	299 (35.9)
Hispanic	382 (45.9)
Black	118 (14.2)
Other	34 (4.1)
**Etiology**	
Alcohol-related	200 (24.0)
Hepatitis B virus	32 (3.8)
Hepatitis C virus	345 (41.4)
MASLD	159 (19.1)
Other	58 (7.0)
**Insurance**	
Medicare	301 (36.6)
Medicaid	45 (5.5)
Parkland Financial Assistance/Jackson card	91 (11.1)
Private	267 (32.5)
Other	84 (10.2)
Uninsured	34 (4.1)
**Annual Household Income***	
<20,000$	153 (18.4)
20,000$-50,000$	181 (21.8)
>50,000$	136 (16.4)
**Child-Pugh Class**	
Child A	454 (55.4)
Child B	268 (32.7)
Child C	97 (11.8)
**Education**	
Less than high school	211 (26.0)
High school grad/GED	259 (31.9)
Some college/technical	212 (26.1)
Bachelors	83 (10.2)

Advanced	47 (5.8)
**Employment**	
Employed	220 (27.1)
Retired	296 (36.5)
Unemployed	105 (12.9)
Disabled	191 (23.5)
**Preferred Language**	
English	502 (60.3)
Spanish	263 (31.6)
English and Spanish equally	67 (8.1)

*Median (25th-75th percentile)

*Income missing in 43% of patients
